# Development of Transcriptomic Resources for Interrogating the Biosynthesis of Monoterpene Indole Alkaloids in Medicinal Plant Species

**DOI:** 10.1371/journal.pone.0052506

**Published:** 2012-12-26

**Authors:** Elsa Góngora-Castillo, Kevin L. Childs, Greg Fedewa, John P. Hamilton, David K. Liscombe, Maria Magallanes-Lundback, Kranthi K. Mandadi, Ezekiel Nims, Weerawat Runguphan, Brieanne Vaillancourt, Marina Varbanova-Herde, Dean DellaPenna, Thomas D. McKnight, Sarah O’Connor, C. Robin Buell

**Affiliations:** 1 Department of Plant Biology, Michigan State University, East Lansing, Michigan, United States of America; 2 Department of Biochemistry and Molecular Biology, Michigan State University, East Lansing, Michigan, United States of America; 3 Department of Chemistry, Massachusetts Institute of Technology, Cambridge, Massachusetts, United States of America; 4 Department of Biology, Texas A&M University, College Station, Texas, United States of America; 5 Department of Biological Chemistry, John Innes Centre, Norwich, United Kingdom; 6 School of Chemistry, University of East Anglia, Norwich, United Kingdom; National Institutes of Health, United States of America

## Abstract

The natural diversity of plant metabolism has long been a source for human medicines. One group of plant-derived compounds, the monoterpene indole alkaloids (MIAs), includes well-documented therapeutic agents used in the treatment of cancer (vinblastine, vincristine, camptothecin), hypertension (reserpine, ajmalicine), malaria (quinine), and as analgesics (7-hydroxymitragynine). Our understanding of the biochemical pathways that synthesize these commercially relevant compounds is incomplete due in part to a lack of molecular, genetic, and genomic resources for the identification of the genes involved in these specialized metabolic pathways. To address these limitations, we generated large-scale transcriptome sequence and expression profiles for three species of Asterids that produce medicinally important MIAs: *Camptotheca acuminata*, *Catharanthus roseus*, and *Rauvolfia serpentina*. Using next generation sequencing technology, we sampled the transcriptomes of these species across a diverse set of developmental tissues, and in the case of *C. roseus*, in cultured cells and roots following elicitor treatment. Through an iterative assembly process, we generated robust transcriptome assemblies for all three species with a substantial number of the assembled transcripts being full or near-full length. The majority of transcripts had a related sequence in either UniRef100, the *Arabidopsis thaliana* predicted proteome, or the Pfam protein domain database; however, we also identified transcripts that lacked similarity with entries in either database and thereby lack a known function. Representation of known genes within the MIA biosynthetic pathway was robust. As a diverse set of tissues and treatments were surveyed, expression abundances of transcripts in the three species could be estimated to reveal transcripts associated with development and response to elicitor treatment. Together, these transcriptomes and expression abundance matrices provide a rich resource for understanding plant specialized metabolism, and promotes realization of innovative production systems for plant-derived pharmaceuticals.

## Introduction

Plants, and natural products derived from them, have been used medicinally for millennia. Even today, over half of the new drugs introduced are natural products or close derivatives, and many of these are from plants [1], [2]. Despite their long history and enduring importance, most medicinal plants have not been bred for increased yield or performance. *Cannabis sativa* is one striking exception, as extensive (but illicit) efforts have increased the concentration of its major active compound, delta 9-tetrahydrocannabinol, from less than 1.5% in the early 1980s to over 13% in more recent samples [3]. This ∼10-fold increase suggests that much potential remains locked in the genomes of other medicinal plants and is waiting to be exploited.

One major barrier to scientifically directed approaches to manipulating the yield and spectrum of pharmacologically active compounds in plants is the limited information on the genes and enzymes that produce these compounds. A further complication is the inability to apply powerful genetic approaches to most medicinal plants–approaches that have had great success in unraveling other complicated biological processes. To circumvent these barriers, we recently participated in the Medicinal Plant Genomics Consortium, an effort to use next-generation sequencing to create public databases for the transcriptomes of 14 medicinal plants (http://medicinalplantgenomics.msu.edu/). The availability of these data allows investigators to freely access information about known biosynthetic genes that are in plants of high pharmacological importance and, importantly, provide the information needed to identify potential genes for the remaining unknown biosynthetic steps for target compounds.

**Figure 1:**
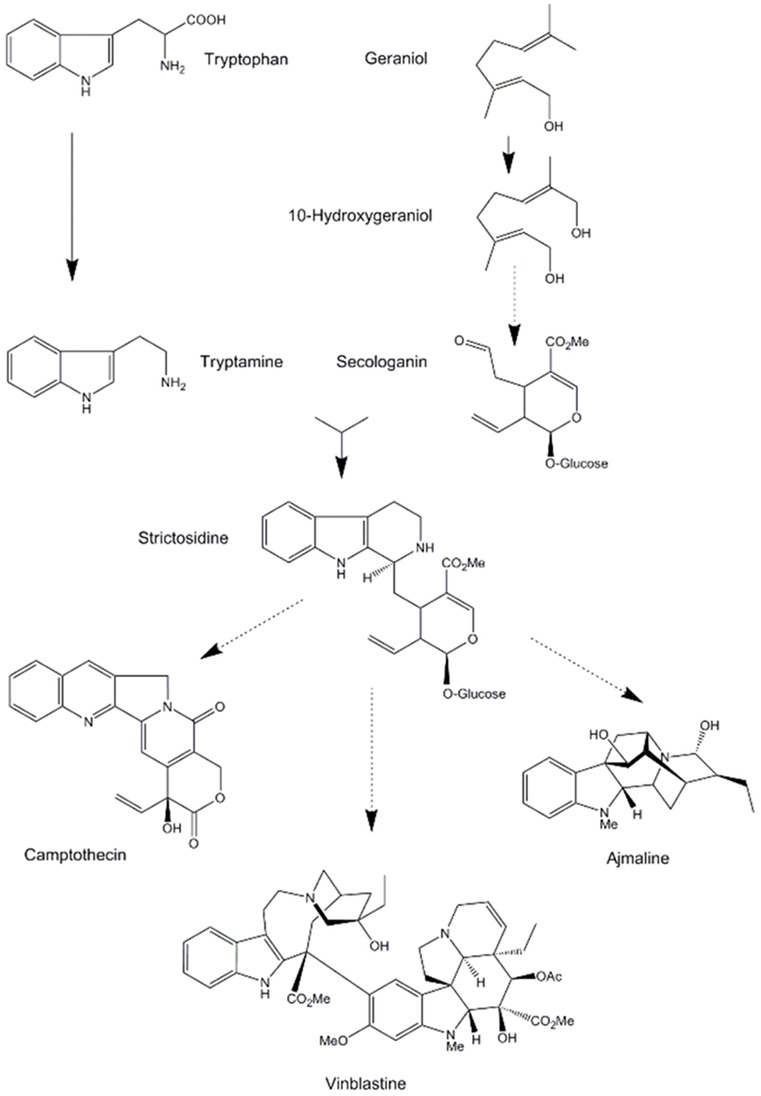
Monoterpene indole alkaloid pathway. The key intermediate strictosidine is formed by condensation of tryptamine, which contributes the indole ring, and secologanin, which is produced from the monoterpene geraniol. In various plants, strictosidine is further metabolized to generate over 2,500 monoterpene indole alkaloids. Solid lines indicate single enzymatic steps; dashed lines indicate multiple steps.

Three asterid species, *Catharanthus roseus* (Apocynaceae), *Rauvolfia serpentina* (Apocynaceae) and *Camptotheca acuminata* (Nyssaceae), produce clinically useful compounds through their monoterpene indole alkaloid (MIA) pathways. The terpenoid portion of these alkaloids is contributed by secologanin, a secoiridoid glycoside produced from geraniol as outlined in Figure 1. Geraniol (as geranylpyrophosphate) also leads to a wide variety of monoterpenes, but it is shunted specifically into the secologanin pathway by geraniol 10-hydroxylase, a P450 oxidase that produces 10-hydroxygeraniol [4], [5]. The indole portion of these alkaloids is contributed by tryptamine, produced by decarboxylation of tryptophan. The first committed enzyme in the monoterpene indole alkaloid pathway is strictosidine synthase, which conjugates secologanin with tryptamine to produce strictosidine. Strictosidine is subsequently modified to produce over 2,500 known MIAs in many plants from several families (for review see [6], [7]). *C. roseus* (Madagascar periwinkle) produces over 100 different indole alkaloids including the clinically important alkaloids vinblastine and vincristine, anticancer agents that act by interfering with microtubule formation [8], [9]. *R. serpentina* (Indian Snakeroot) produces more than 50 different indole alkaloids including ajmaline, an indole alkaloid used to diagnose and treat ventricular arrhythmias [10], as well as reserpine, an antihypertensive alkaloid [11]. *C. acuminata* (Chinese Happy Tree) produces dozens of indole and indole-derived quinoline alkaloids including camptothecin, a powerful inhibitor of DNA topoisomerase I and the starting compound for semi-synthetic antitumor drugs including topotecan, irinotecan, and belotecan [12], [13]. Although the indole ring is rearranged to a quinoline ring in camptothecin, we include it here as its biosynthetic pathway is shared with bona fide MIAs up to strictosidine.

Here, we describe results from transcriptomic analysis of *C. acuminata*, *C. roseus,* and *R. serpentina* that entailed a broad survey of transcripts in tissues, organs, and treatments to capture the breadth and depth of these transcriptomes. As we coupled high throughput next generation sequencing methods with computational approaches, we were able to determine expression abundances of representative transcripts in a diverse set of conditions for each of the species. In addition to providing a representative transcriptome and expression atlas for each of these species, we performed comparative analyses to identify orthologous transcripts within these three asterids to begin to identify conserved and diverged genes involved in MIA biosynthesis.

## Results and Discussion

### Sequencing and Transcriptome Assembly

Our approach to generate a robust set of transcript assemblies for these three species was to isolate RNA from a broad range of cell types (Table S1), construct normalized and non-normalized cDNA (experimental) libraries, and generate paired and single end sequences using the Illumina sequencing platform. Libraries were sequenced (36–120 nucleotides) in the single or paired end mode (Tables 1, 2, 3) in a single or multiple lanes, generating 16.9 to 129.5 million purity filtered reads per library. Overall, reads were of high quality with an average 74.8% passing the Illumina purity filter.

**Table 1:** Libraries and sequencing generated for *Camptotheca acuminata*.

Tissues	Reads	Read Length (bp)	No. Reads (M)	No. Purity Filtered Reads(M)	Percentage of Purity Filtered Reads
**Developmental**					
Lower bark^1^	SE	36	19.2	16.9	88.0%
Upper bark^2^	PE	76	100.4	84.5	84.2%
Immature leaf^1^	SE	36	20.2	17.7	87.6%
Mature leaf, replicate 1^3^	PE	76	116.8	90.8	77.7%
Mature leaf, replicate 2^3^	SE	36	69.5	58.2	83.7%
Trichomes^1^	PE	76	93.7	81.4	86.9%
Entire root^3^	PE	76	113.6	88.9	78.3%
Immature flower^3^	PE	76	82.6	72.7	88.0%
Immature fruit^2^	PE	76	100.3	85.2	84.9%
Mature fruit^3^	PE	76	56.3	50.2	89.2%
Whole seedlings^2^	PE	76	47	40.4	86.0%
Cotyledons^3^	PE	76	84.8	74.1	87.4%
Seedling apical stem^2^	PE	76	88.3	76.1	86.2%
Seedling basal stem^3^	PE	76	83.8	72.6	86.6%
Seedling leaf^1^	PE	76	99.1	83.9	84.7%
Seedling lateral roots^2^	PE	76	109.2	88.9	81.4%
Seedling tap roots^2^	PE	76	68	61.7	90.7%
**Cultured cells/tissues**					
Callus^1^	SE	36	38.8	24.9	64.2%
Root culture^2^	SE	36	38.8	25.8	66.5%
**Total Reads (M)**			1430.4	1194.9	

1 Libraries used in the first, initial de novo assembly.

2 Libraries only sequenced for expression abundance estimates, reads were not included in de novo assembly.

3 Libraries used in the second, final de novo assembly.

M: Million.

**Table 2:** Libraries and sequencing generated for *Catharanthus roseus*.

**Tissues**	**Library**	**Read Length (bp)**	**No. Reads (M)**	**No. Purity Filtered Reads (M)**	**Percentage of Purity Filtered Reads**
**Developmental**					
Sterile seedlings	SE	36	32.9	26.7	81.2%
Mature leaf	SE	36	30	25.3	84.3%
Immature leaf	SE	36	38.4	29.9	77.9%
Stem	SE	36	38.1	28.9	75.9%
Root	SE	36	41.1	30.8	74.9%
Flowers	SE	36	43.5	31.6	72.6%
**Elicitor Treatment** 1					
Sterile seedlings MJ 0 h	SE	36	23.9	21.2	88.7%
Sterile seedlings MJ 6 h	SE	36	48.1	24.7	51.4%
Sterile seedlings MJ 12 h	SE	36	46.9	30	64.0%
Sterile seedlings MJ 24 h	SE	36	33.9	27.5	81.1%
Sterile seedlings MJ 5 d	SE	36	42.4	30.9	72.9%
Sterile seedlings MJ 12 d	SE	36	35.1	28.7	81.8%
**Cultured Cells/Tissues** 2					
Suspension culture YE 6 h	SE	36	34.5	28.1	81.4%
Suspension culture YE 12 h	SE	36	42	31.7	75.5%
Suspension culture YE 24 h	SE	36	34.3	28	81.6%
Suspension culture (control)	SE	36	48.7	26.2	53.8%
WT Hairy roots	SE	36	29	25.3	87.2%
WT Hairy roots MJ 0 h	SE	36	40.7	17	41.8%
WT Hairy roots MJ 24 h	SE	36	42.4	19.1	45.0%
Tryptophan decarboxylase silenced Hairy root	SE	36	25.9	22.8	88.0%
Tryptophan decarboxylase silenced Hairy roots - MJ 0 h	SE	36	41.6	20.2	48.6%
Tryptophan decarboxylase silenced Hairy roots MJ 24 h	SE	36	45.5	21.8	47.9%
Halogenase expressing Hairy root	SE	36	25.1	22	87.6%
**Pooled Samples**					
Normalized library	PE	120	80.1	66.6	83.1%
Normalized library	PE	55	137.1	117.7	85.8%
**Total Reads (M)**			1048.3	756	

1 Sterile seedlings were treated with methyl jasmonate (MJ, 6 or 100 µM).

2 Suspension culture cells were treated with yeast extract (YE,0.3 mg/mL). Hairy roots were treated with methyl jasmonate (MJ, 250 µM).

M: Million.

**Table 3:** Libraries and sequencing generated for *Rauvolfia serpentin*a.

Tissues	Library	Read Length (bp)	No. Reads (M)	No. Purity Filtered Reads (M)	Percentage of Purity Filtered Reads
Mature leaf	SE	36	36.9	24.5	66.4%
Young leaf	SE	36	35	26.6	76.0%
Pigmented portion of stem	SE	36	36.5	27.9	76.4%
Upper stem	SE	36	39.5	19.9	50.4%
Woody stem	SE	36	38.5	25.1	65.2%
Young root	SE	36	43.7	20.1	46.0%
Mature root	SE	36	37.1	28.2	76.0%
Flower	SE	36	35.9	28	78.0%
Pooled Samples (Normalized library)	PE	120	154.2	129.5	84.0%
**Total Reads (M)**			457.3	329.8	

M: Million.

Due to the computational limitations in *de novo* assembly of transcriptomes with short read sequences, an initial *de novo* assembly using a representative set of transcript reads was made. The initial transcriptome was then used to filter out redundant transcripts in additional libraries to identify novel reads for inclusion in a second and final *de novo* assembly. The initial *de novo* assemblies of *C. roseus* and *R. serpentina*, were made using the transcriptome assembler Oases [14] with reads from normalized cDNA libraries in which RNA from multiple tissues were pooled in an equimolar manner and used to make a single normalized library that was sequenced in the paired end mode. The longest transcript isoform generated from each Oases-generated contig was selected to be the representative transcript and an artificial molecule (pseudomolecule) was constructed using custom Perl scripts for *C. roseus* and *R. serpentina* by concatenating the representative transcripts together. Pre-processed reads from experimental libraries, derived from single tissues or treatments (Table 2 and 3), i.e., non-normalized, were then mapped to the pseudomolecule using the short read aligner TopHat [15]; reads that failed to align, i.e., novel relative to the assembled transcriptome, were retained. The second and final *de novo* assembly for *C. roseus and R. serpentina* was generated by combining reads from the normalized library with reads that failed to align to the pseudomolecule from the single non-normalized experimental tissue/treatment libraries and using these in a second and final assembly with Oases [14]. For *C. acuminata*, the initial *de novo* assembly was generated using sequences from libraries constructed from five individual tissues (lower bark, immature leaf, seedling leaf, trichomes and callus) and a pseudomolecule was constructed using the representative transcript from each contig. Reads from seven other single tissue libraries (Table 1) were then mapped to the pseudomolecule and non-aligned reads were retained and used with reads from the five initial libraries to generate the second and final *de novo C. acuminata* assembly.

**Figure 2:**
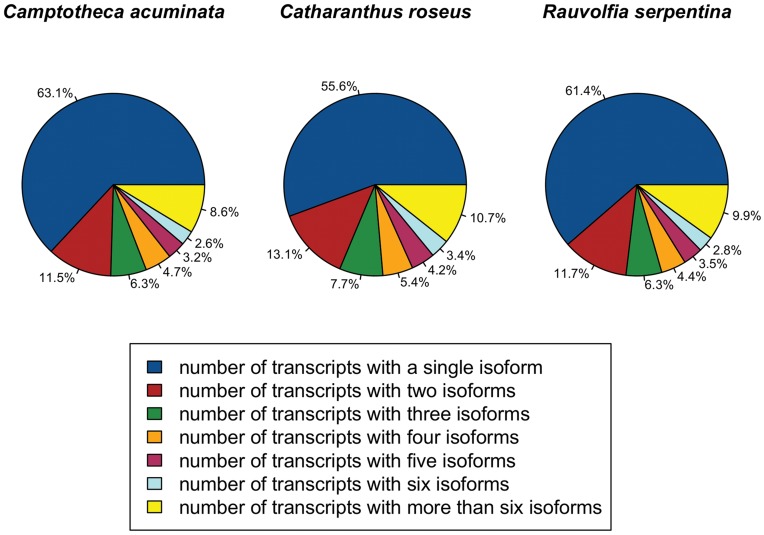
Frequency of Oases-derived isoforms in transcript assemblies of *Camptotheca acuminata*, *Catharanthus roseus* and *Rauvolfia serpentina.* The proportion of transcripts with single and multiple isoforms in each of the three species.

As shown in Table 4, 69.0–81.8% of the reads were used in the final assembly across the three species generating robust transcriptome assemblies for each species as shown by the N50 contig size which ranged from 1,628 bp to 1,722 bp. The final set of transcripts were examined for low complexity by filtering out assembled sequences with large sub-sequences of homopolymers and contamination with microbial, fungal, and/or insect sequences using BLASTX [16] searches against UniRef100 [17]; only a small number of these sequences were removed from the datasets due to potential contamination and/or low quality contigs (*C. acuminata*: 1,313, *C. roseus*: 816, *R. serpentina*: 698). The transcriptome assembler Oases [14] identifies isoforms, which in the case of heterozygous diploid species may be true alternative splice variants, alleles, or close paralogs. In the *C. acuminata*, *C. roseus*, and *R. serpentina* transcriptome assemblies, we identified a total of 121,109, 86,725, and 99,637 transcripts, respectively. The majority of transcripts were present as a single isoform (Figure 2), however, a subset of transcripts did have more than one isoform and collectively, we identified 53,154, 32,607, and 41,405 unique transcripts or unigenes (see Materials and Methods) in *C. acuminata*, *C. roseus*, and *R. serpentina*, respectively (Table 4).

**Table 4:** Summary of statistics of the transcriptome de novo assemblies of *Camptotheca acuminata*, *Catharanthus roseus*, and *Rauvolfia serpentina*.

	*C. acuminata*	*C. roseus*	*R. serpentina*
Total Reads used for assembly	177,515,831	192,869,864	93,142,503
Total Reads in assembly (Percentage)	122,518,542 (69.0%)	138,212,150 (71.6%)	76,282,802 (81.8%)
			
N50 (nt)	1,722	1,679	1,628
Average contig size (nt)	1,197	1,236	1,201
Min contig size (nt)	251	251	251
Max contig size (nt)	15,958	12,048	7,943
GC content	42.55%	40.68%	42.04%
Total transcript number	121,109	86,725	99,637
Contig with 1 transcript isoform	33,749	21,298	25,481
Contig with >1 transcript isoforms	19,405	11,309	15,924
Total number unique transcripts	53,154	32,607	41,405
Median isoform number for contigs with >1 transcript isoform	4	4	4

### Annotation of the Transcriptome Assemblies

**Figure 3:**
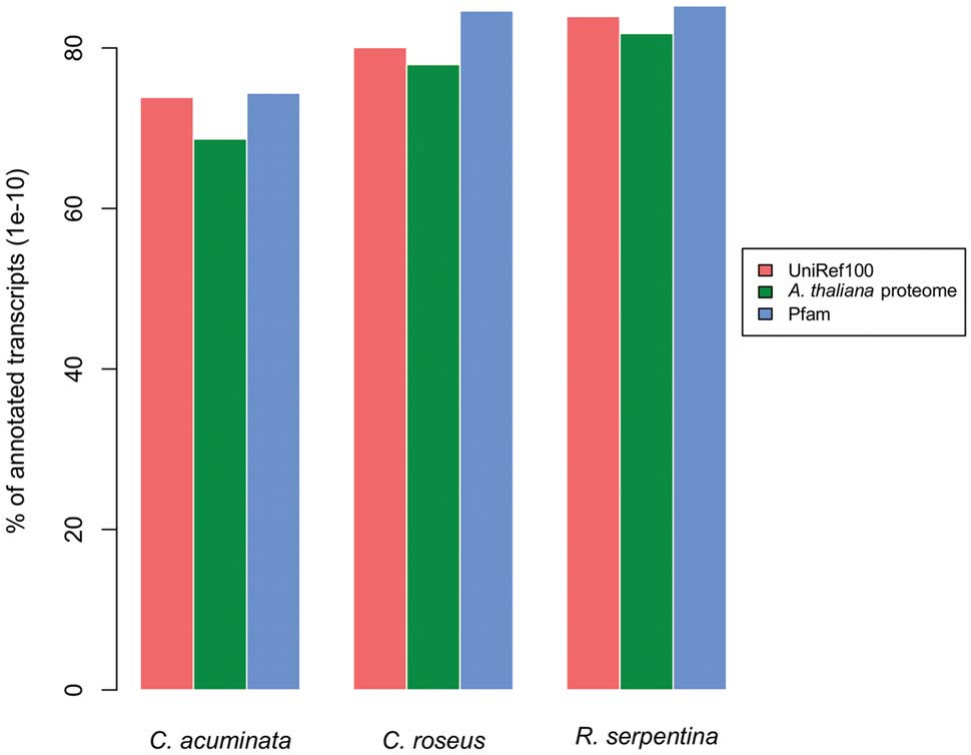
Functional annotation results. Proportion of *Camptotheca acuminata*, *Catharanthus roseus* and *Rauvolfia serpentina* transcripts with sequence similarity to the UniRef100 database [17], *Arabidopsis thaliana* proteome (http://arabidopsis.org), and Pfam domain database [18].

**Figure 4:**
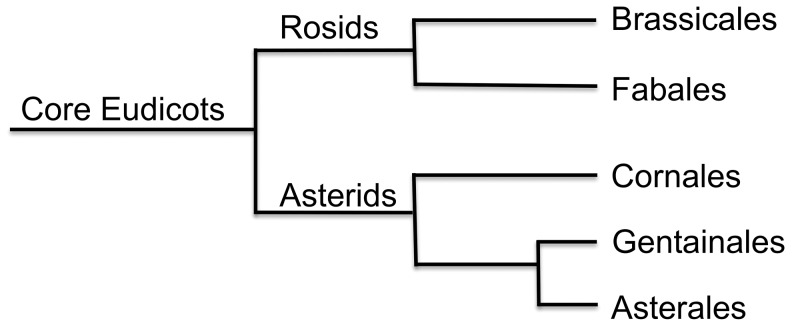
Phylogenetic relationships. *Camptotheca acuminata* (Nyssaceae) is in the order Cornales within the asterid superorder of core eudicots, and *Catharanthus roseus* and *Rauvolfia serpentina* (both Apocynaceae) are in Gentainales, also within the asterids. *Arabidopsis thaliana* is in the family Brassicales within the rosid superorder. Fabales and Astrales are shown for orientation. Redrawn and greatly simplified from APG III [19].

The final transcriptome assemblies for the three species were annotated using a combination of evidence from sequence similarity searches to the UniRef100 database [17] and the predicted *Arabidopsis thaliana* proteome (TAIR10, http://arabidopsis.org), as well as Pfam domain [18] composition. As shown in Figure 3, all three transcriptomes had similar proportions of sequences with similarity to the UniRef100 database although *C. roseus* (80.1%) and *R. serpentina* (83.9%) had a higher proportion of their transcriptomes with similarity to UniRef100 entries compared to *C. acuminata* (73.8%). This was mirrored in the occurrence of Pfam domains among the transcriptomes, ranging from 74.4% in *C. acuminata* to 84.5% in *R. serpentina*. An analogous pattern was observed in similarity to the predicted *A. thaliana* proteome (Figure 3); the proportion of sequences from each transcriptome with similarity to *A. thaliana* was 77.9%, 81.8%, and 68.6% for *C. roseus, R. serpentina*, and *C. acuminata*. Phylogeny does not explain this pattern, as all three indole alkaloid species are asterids and therefore evolutionarily equidistant from *A. thaliana*, a rosid within the Brassicales (Figure 4). However, *C. roseus* and *R. serpentina* are closely related, as they both are within the Apocynaceae family (order Gentianales), whereas *C. acuminata* is in the Nyssaceae family (order Cornales) [19].

To more broadly compare the representation of our assemblies to each other and how they represent a prototypic plant transcriptome, we annotated the transcripts with Gene Ontology (GO) Slim terms [20] and compared these to the complete *A. thaliana* proteome. In total, GO Slim terms were assigned to 11,223, 10,603 and 13,639 of the representative transcripts in *C. acuminata*, *C. roseus* and *R. serpentina,* respectively. When compared to the set of GO Slim terms in the full *A. thaliana* proteome, similar representations of the molecular function, biology process, and cellular component categories were apparent in our transcript assemblies (Figure S1). It is important to note that the representation of GO associations in our transcript datasets may over- or under-represent the actual gene complement in *C. acuminata*, *C. roseus* and *R. serpentina.* Extremely lowly expressed genes will not be present in our assemblies while categories in which there are a high number of alleles or isoforms that are not within a single Oases-generated contig will artificially inflate representation of that GO Slim category.

We further characterized our assemblies by searching each assembly against known mRNA and peptide sequences available for each species in GenBank. For *C. acuminata*, 52 nuclear-encoded peptide sequences were available in GenBank, and we were able to detect 42 (80.7%) in our assembly. The forty-two detected transcripts were full-length or near full-length (with greater than 80% coverage). For *R. serpentina*, 74 nuclear-encoded peptide sequences were available in GenBank, and we were able to detect all 74 in our assembly. All of these were full-length or near full-length (with greater than 80% coverage). Substantially more sequences were available for *C. roseus* as a set of 19,886 Sanger-generated Expressed Sequence Tags (ESTs) available in dbEST. We were able to identify 9,287 of these ESTs in our *C. roseus* transcriptome. Of the 362 documented *C. roseus* nuclear-encoded peptide sequences in GenBank, we were able to identify 290 (80.1%), and all of these were full-length or near full-length (with greater than 80% coverage), further confirming the robust representation of the *C. roseus* transcriptome in our assembly.

### Expression Abundances and Relationship of Tissues

**Figure 5:**
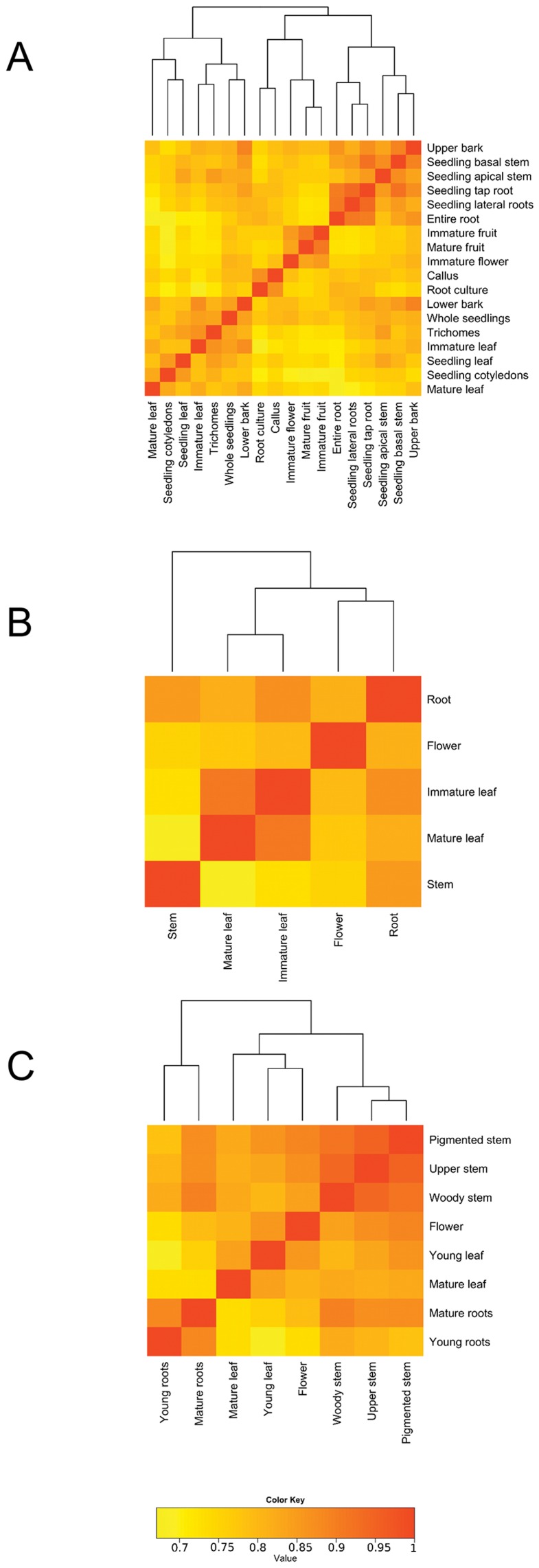
Hierarchical clustering of expression profiles from sampled tissues from *Camptotheca acuminata* (A), *Catharanthus roseus* (B), and *Rauvolfia serpentina* (C). Pearson product-moment correlation coefficient of log_2_ FPKM (fragments per kb transcript per million mapped reads) expression values among RNA-seq libraries were calculated and clustered using R [39]; negative values were set to zero.

No genome sequence is available for *C. acuminata*, *C. roseus* and *R. serpentina,* and thus pseudomolecules constructed from the representative transcripts derived from the final *de novo* assemblies were used as a reference to determinate the transcript abundances across our tissues and treatments. To quantify the expression levels of each transcript within each library, we mapped the reads from all experimental libraries to their pseudomolecule using TopHat [15] and quantified expression abundances using Cufflinks [21], [22]. Expression levels, represented as fragments per kilobase of transcript per million mapped reads (FPKM) for all libraries (Table 1, 2, 3), except the normalized libraries from *C. roseus* and *R. serpentina,* were generated (Tables S2, S3, S4). Not all reads within a library were incorporated into the assembly and thus expression abundances could only be calculated for those transcripts represented in the final *de novo* assembly. Furthermore, reads that map to multiple locations within the overall set of representative transcripts are penalized within the Cufflinks algorithm with respect to FPKM estimations [21]. However, hierarchical clustering of the expression levels in developmental tissues from each species show correlated gene expression in similar tissues (Figure 5A–C), confirming our approach to estimation of transcript abundances.

### Orthologous and Paralogous Clustering

**Figure 6:**
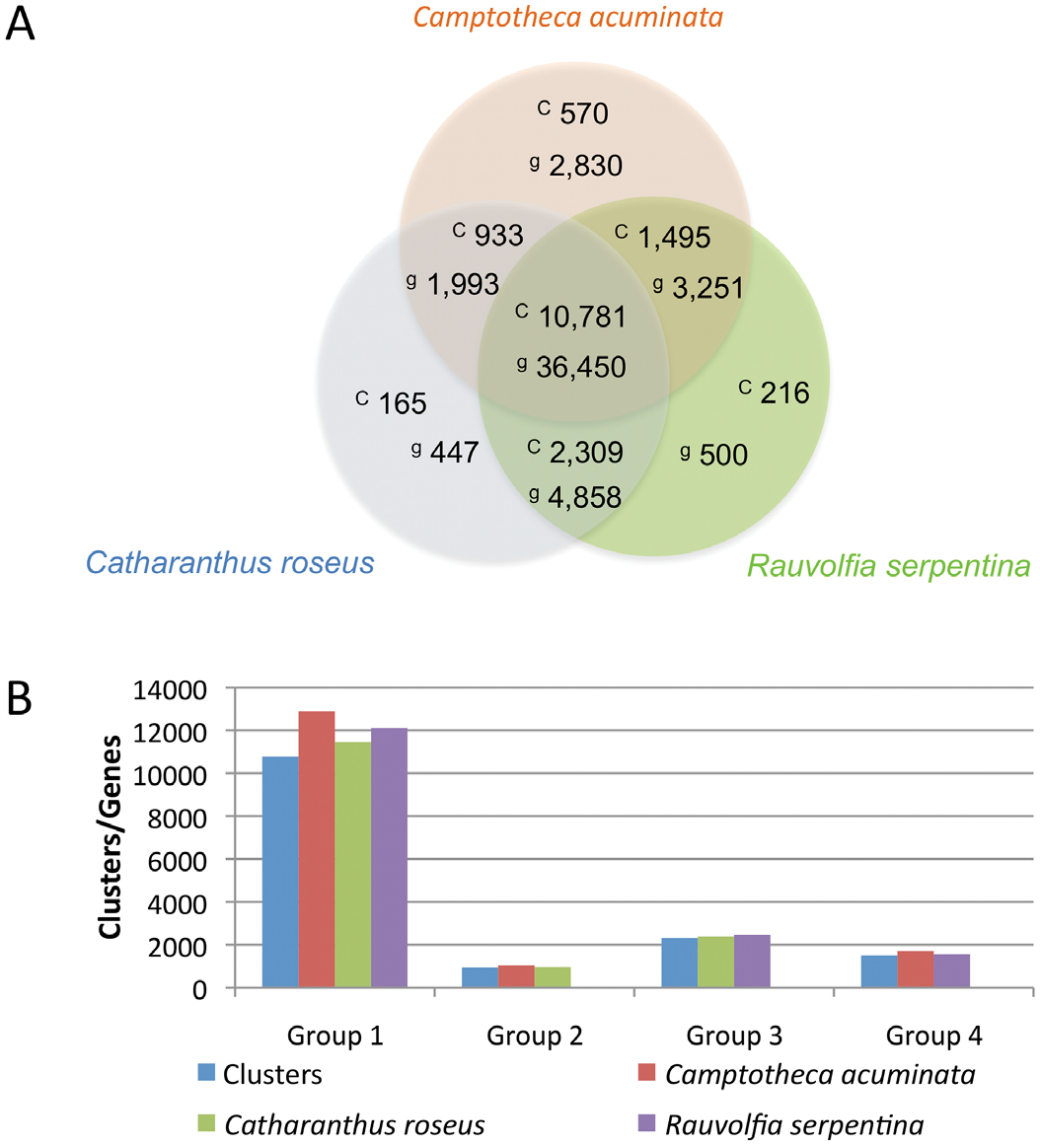
Cluster of orthologous and paralogous genes families in *Camptotheca acuminata*, *Catharanthus roseus,* and *Rauvolfia serpentina* species as identified by OrthoMCL. Predicted peptides from the *Camptotheca acuminata*, *Catharanthus roseus* and *Rauvolfia serpentina* transcriptomes were clustered using OrthoMCL [23]. A) Number of clusters (c) and genes (g) for each orthologous group. B) Number of genes in the different clusters for each species. The number of clusters and genes for each OrthoMCL group are shown. Group 1: Clusters (blue) and genes shared among *C. acuminata* (red), *C. roseus* (green) and *R. serpentina* (purple). Group 2: Clusters (blue) and genes shared among *C. acuminata* (red) and *C. roseus* (green). Group 3: Clusters (blue) and genes shared among *C. roseus* (green) and *R. serpentina* (purple). Group 4: Clusters (blue) and genes shared among *C. acuminata* (red) and *R. serpentina* (purple).

All three species are asterids and most likely share a large set of transcripts that encode proteins responsible for cell function. Using the OrthoMCL algorithm [23], we generated clusters of transcripts that encode orthologs and close paralogs (Figure 6A). A total of 10,781 clusters containing 36,450 transcripts (Table S5) from all three species were identified, consistent with previous reports that there are a core set of genes common to all plant species [24], [25]. However, we were able to identify transcripts shared between only two species as well as transcripts unique to each species. More transcripts were found in the *C. roseus-R. serpentina* cluster (Figure 6B, Table S6; 2,309 clusters containing 4,858 transcripts) than the *C. acuminata-C. roseus* (Figure 6B, Table S7; 933 clusters, 1993 transcripts) or the *C. acuminata-R. serpentina* (Figure 6B, Table S8; 1,495 clusters containing 3,251 transcripts) clusters, consistent with the close phylogeny of *C. roseus* and *R. serpentina* within the Apocynaceae family. The OrthoMCL algorithm also identifies close paralogs within a species and we identified 570, 165 and 216 clusters restricted to *C. acuminata, C. roseus*, and *R. serpentina*, respectively (Tables S9, S10, S11). As the Oases-derived transcript assemblies may not be able to distinguish alternative splice forms from alleles and close paralogs, these clusters may represent one or more of these transcript variants sampled and generated in our assembly. Not all transcripts could be clustered with the OrthoMCL algorithm; 12,913, 10,110 and 16,890 transcripts from *C. acuminata, C. roseus* and *R. serpentina*, respectively could not, therefore remaining as singleton transcripts (data not shown).

### Expression of Monoterpene Indole Alkaloid Biosynthetic Pathway Genes

**Figure 7:**
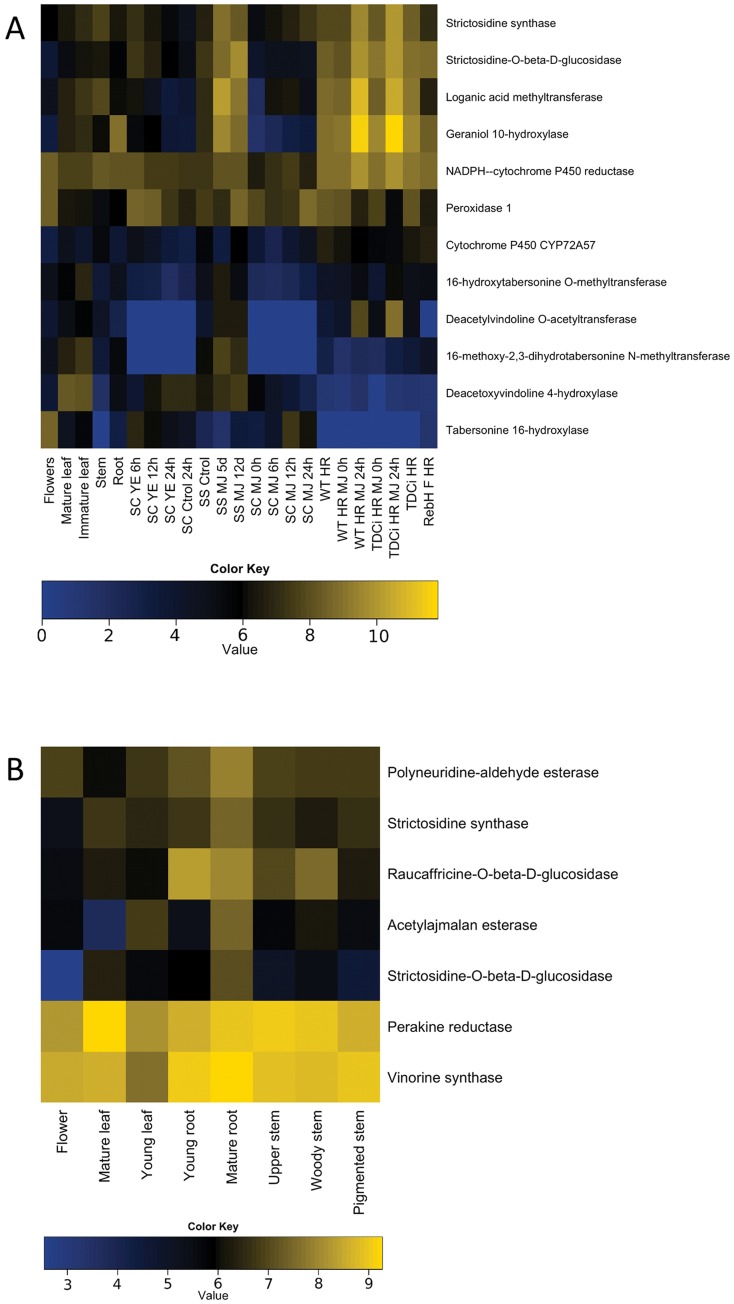
Expression patterns of known genes in monoterpene indole alkaloid biosynthesis across different tissues and treatments. Expression values in log_2_ FPKM (fragments per Kilobase of transcript per million fragments mapped) were calculated, negative values were set to zero and then were clustered using R [39]. A) *Catharanthus roseus*: Expression values were sorted in three major groups: Developmental tissues, Yeast extract (YE) treatment of suspension cells (SC), and Methyl jasmonate (MJ) treatment of sterile seedlings (SS) and hairy roots (HR). B) *Rauvolfia serpentina*. Expression values shown represent the different developmental tissues.

We examined the representation of known, validated genes in the MIA biosynthetic pathway in each species. For *C. roseus*, we were able to find all 12 of the known and validated genes [26] involved in the biosynthesis of vinblastine. Using our FPKM values, we examined expression of these genes across the set of tissues and treatments sampled in this study (Figure 7A). Notably, alkaloid biosynthetic genes were up-regulated in response to methyl jasmonate, an elicitor that has been shown to improve alkaloid production in *C. roseus* [27]. For *R. serpentina*, we could identify all seven of the known and validated biosynthetic pathway genes [26]. Expression abundances across the six sampled *R. serpentina* tissues for these genes were coordinated (Figure 7B), which bodes well for the successful identification of the remaining pathway genes by coexpression analyses. The representation of the known biosynthetic genes in the *C. roseus* and *R. serpentina* transcriptome assemblies testifies to the quality of the transcriptomic data and the prospects for these data sets to serve as a resource for mining undiscovered pathway genes.

Tryptophan decarboxylase 1 and 2 are the only two genes that have been experimentally shown to be involved in camptothecin biosynthesis [28]; both of which were found in our *C. acuminata* transcriptome assembly (transcript ID 5306 and 20066). However, using sequence similarity searches, we were able to find candidate genes in *C. acuminata* that encode early steps in the pathway identified in other monoterpene indole alkaloid producing plants: Geraniol 10-hydroxylase (transcript ID 42007), 1-deoxy-D-xylulose-5-phosphate synthase (transcript ID 1818), 1-deoxy-D-xylulose-5-phosphate reductoisomerase (transcript ID 2329), 4-diphosphocytidyl-2-C-methyl-D-erythritol kinase (transcript ID 8816), 2-C-methyl-D-erythritol 2,4-cyclodiphosphate synthase (transcript ID 3259), 4-Diphosphocytidyl-2C-methyl-D-erythritol synthase (transcript ID 13238), 4-hydroxy-3-methylbut-2-enyl diphosphate synthase (transcript ID 2848) and 4-hydroxy-3-methylbut-2-enyl diphosphate reductase (transcript ID 13145 and 1619). As there are no known genes encoding biochemically confirmed proteins after geraniol-10-hydroxylase and tryptophan decarboxylase in *C. acuminata*, it is difficult to assess the potential for coexpression analysis in identifying candidate pathway genes in this species.

### Conclusions

Here, we report transcriptomic data sets for three medicinal plants that produce structurally diverse, yet biosynthetically related monoterpene indole alkaloids. We believe that these data sets, both individually and collectively, will enable the discovery of new biosynthetic genes involved in the production of medicinally important monoterpene indole alkaloids across these and other species. The assemblies provide the near to full-length sequence of these genes to enable their efficient cloning and expression without resorting to rapid amplification of cDNA end methods. Bioinformatic strategies such as co-expression analysis can be applied to the expression matrices to identify new candidate genes involved in metabolic pathways, which has been show already with the *C. roseus* data set described in this study [29], [30]. The capability of next-generation sequencing to rapidly and inexpensively provide a near-complete transcriptome now opens the door to elucidating some of the most chemically prolific, but genetically intractable, species of plants.

## Materials and Methods

### Germplasm, Growth Conditions and Tissues Used in this Study

*C. acuminata* plants were grown at Michigan State University (MSU, 47.2 latitude, −84.5 longitude), Texas A&M University (TAMU, 30.6 latitude, −96.3 longitude) and the Thad Cochran Natural Products Center in Oxford, Mississippi (34.4 latitude, −89.5 longitude). The latter site has mature established trees grown under natural conditions. All plants grown at MSU and TAMU were derived from seed collected from a single *C. acuminata* tree at the San Antonio Zoo (29.4 latitude, −98.5 longitude). *C. acuminata* plants grown in MSU growth chambers were cultivated at 100–150 microeinsteins from a combination of fluorescent and incandescent lights with a 14-hour day (21°C) and 10 hr night (18°C). For greenhouse growth at MSU, tissue culture grown plants with 4–8 leaves were transferred to large pots containing a 1∶1∶1 mixture of Redi-Earth:Perlite:coarse vermiculite and Osmocote (13∶13∶13) slow release fertilizer. Greenhouse plants were grown under natural light with approximately 200 microeinstein of supplemental light from sodium lamps to maintain a minimum 12 hr day. The temperature was maintained at 30°C during the day and 22°C at night. Whole tissues were harvested as described in Table S1. Sterile culture of roots and callus was performed on ‘CacA’ medium (MS salts, 4.4 g/L; pH 6.5; B5 vitamins 1.0 ml/L (1000X stock); NAA 4.0 ml/L (4 mg/L stock); BA 2.0 ml/l (2 mg/L stock); sucrose 100 ml/L (30% stock)).

*C. roseus* plants were grown at the Massachusetts Institute of Technology (Latitude: N 42° 21′ 35.1248′′ Longitude: W 71° 5′ 36.8135′′.) Seeds were obtained from B&T World Seeds (Paguignan, France; Little Bright Eyes). *C. roseus* plants were germinated and grown in Miracle-Gro potting mix grown at ambient temperature (18–21°C). Full spectrum sunlamps were used at 100–150 microeinsteins with a 14-hour day and 10 hr night. Tissues were harvested as described in Table S1. Hairy root cultures were obtained by infection with *Agrobacterium rhizogenes* as previously described [31], [32], [33]. Cell suspension culture was obtained from callus purchased from Leibniz Institute DSMZ-German Collection of Microorganisms and Cell Cultures (catalogue number PC510). Temperature was maintained at 30°C during the day and 22°C at night. Sterile seedlings were cultured on plates in MS with 1% agar. Hairy roots were cultivated in the dark in Gamborg’s B5 media (half strength basal salts, full strength vitamins, 30 g sucrose L-1, pH 5.7) and recultured every two weeks at 125 rpm at room temperature (25°C). Cell suspension cultures were grown at 125 rpm at room temperature (25°C) on a 12-hour light, 12-hour dark cycle in AM1B media (PhytoTechnology Laboratories, Shawnee Mission, Kansas). Cultures were recultured every two weeks.

*R. serpentina* plants were grown at the Massachusetts Institute of Technology (Latitude: N 42° 21′ 35.1248′′ Longitude: W 71° 5′ 36.8135′′). Seeds were obtained from Richter’s (Goodwin, Ontario, Canada), germinated and grown in Miracle-Gro potting mix grown at ambient temperature (18–21°C). Full spectrum sunlamps were used at 100–150 microeinsteins with a 14-hour day and 10 hr night. Tissues were harvested as described in Table S1.

### RNA Isolation, Library Construction, and Sequencing

RNA was isolated as described previously [22]. Normalized cDNA libraries were constructed from RNA from single reference plants for *C. roseus* and *R. serpentina*. For *C. roseus*, equimolar RNA from mature leaf, immature leaf, flower, upper stem, and entire root were pooled and mature leaf, stem, root and flowers were similarly pooled for *R. serpentina*. Normalized cDNA libraries were constructed using double stranded nuclease (Evrogen, Russia), adaptors used in the normalization process were removed by restriction with *Sfi*I prior to use in Illumina library construction. Two types of libraries were constructed for Illumina sequencing. For normalized cDNA, the Illumina Paired End DNA library construction kit (Illumina, San Diego, CA) was used. Fragment sizes for *C. roseus* were on average 407 bp whereas fragment sizes for *R. serpentina* averaged 422 bp. For *C. acuminata*, initial quality assessments with a normalized cDNA library revealed contamination and it was discarded. Thus, libraries from single RNA samples were constructed for *C. acuminata*; the average fragment size of the *C. acuminata* paired end libraries was 337 bp. All libraries were sequenced on the Illumina platform at the MSU Research Technology Support Facility or at the FastTrack Sequencing Facility at Illumina (San Diego, CA). Libraries were sequenced in the single and/or paired end read mode with lengths ranging from 36 bp to 120 bp (Tables 1, 2, 3). Sequences are available in the NCBI Sequence Read Archive under accession numbers SRP006028 (*R. serpentina*), SRP005953 (*C. roseus*) and SRP006330 (*C. acuminata*).

### Transcriptome Assembly and Expression Abundances

Reads were cleaned and filtered for quality as described previously [22]. For *C. roseus* and *R. serpentina*, reads from the normalized libraries were used in a *de novo* assembly using Velvet (v 1.0.17) [34] and Oases (v 0.1.18) [14]. The initial *de novo* assembly of the *C. acuminata* transcriptome was generated by assembly of reads from seedling leaf, trichome, lower bark, immature leaf, and callus libraries. The initial *de novo* assembly was built using a k-mer of 31 for each species. Custom Perl scripts were used to construct an artificial genome (pseudomolecule) for each species using a representative transcript from the Oases-generated contigs [22]. For *C. roseus* and *R. serpentina*, novel reads absent in the initial *de novo* assembly were identified in the non-normalized read datasets by mapping single-end reads from each species to their cognate pseudomolecule using TopHat (v 1.2.0) [15]. For *C. acuminata*, novel reads absent in the initial *de novo* assembly were identified in seven other single tissue libraries (mature leaf (both replicates), entire root, immature flower, mature fruit, seedling cotyledons and seedling basal stem) by mapping reads to the *C. acuminata* pseudomolecule using TopHat (v 1.2.0) [15]. Novel reads were combined along with the initial reads utilized in the first *de novo* assembly to make a final *de novo* assembly. A range of *k*-mers was tested for each assembly and an optimal *k*-mer of 31, 27 and 27 was found for *C. acuminata*, *C. roseus* and *R. serpentina*, respectively. Assembled transcripts can be downloaded at http://medicinalplantgenomics.msu.edu/and have been deposited at DDBJ/EMBL/GenBank under the accession numbers GACD00000000 (*C. roseus*), GACE00000000 (*R. serpentina*), and GACF00000000 (*C. acuminata*). The versions described in this paper are the first versions: GACD01000000 (*C. roseus*), GACE01000000 (*R. serpentina*), and GACF01000000 (*C. acuminata*). To conform to the NCBI standards, all sized gaps within the Oases assemblies were converted to a gap with 10 Ns. The unique transcript set (or unigene set) was defined as all of the single isoform transcripts plus a representative transcript (the longest transcript) from contigs in which more than one isoform was generated by Oases [14]. Following construction of a second pseudomolecule, all reads from all libraries (except the normalized cDNA libraries) were mapped using TopHat (v 1.2.0) [15] and transcript abundances expressed in FPKM as determined using Cufflinks (v 0.9.3) ([21], Tables S2, S3, S4, http://medicinalplantgenomics.msu.edu/).

### Annotation of Transcripts

Assembled transcripts were annotated using BLASTX searches against UniRef100 [17] and transcripts that had a hit with ≥70% identity and over ≥90% coverage to bacterial, fungal, viral, viroid, arthropod, stramenopile or human sequences were removed as contaminants. Predicted translations were generated using ESTScan (v 3.0.3) [35] and Pfam domains assigned using HMMER (v 3.0) and Pfam 24.0 [18]. Functional annotation was assigned to the assembled transcripts using a combination of UniRef100 matches and Pfam domain results (Tables S2, S3, S4). An E-value cutoff of 1e-10 was used to assign significant similarity. Transcripts were also searched against the *A. thaliana* proteome (TAIR10, http://arabidopsis.org) and against peptides and ESTs deposited in GenBank for these three species through BLASTX and BLASTN searches, respectively [16]. GenBank sequences dataset were pre-processed using custom Perl script, and peptides and ESTs less than 100 amino acids (or nucleotides for the ESTs) were filtered out. GO associations [36] were made by InterProScan (v 4.6) [37]; GO terms were further reduced to GO Slims terms using custom Perl scripts.

### Computational Analyses

Orthologs and close paralogs were identified in the three predicted proteomes using OrthoMCL (v 1.4) [23], [38] using the default parameters with an E-value cutoff of 1e-10. To reduce false grouping of paralogs due to alternative isoforms, a representative transcript defined as the model that produces the longest peptide was used in the orthologous clustering. Transposable elements were filtered out to avoid clusters comprised entirely of transposable elements. Correlation of expression profiles among the tissues was calculated by the Pearson product-moment correlation coefficient of log2 FPKM using R [39], negative values were set to zero.

## Supporting Information

Figure S1 **Gene Ontology functional annotation.** Assignment of Gene Ontology Slim (GOSlim) terms to the *Arabidopsis thaliana* proteome, and the representative transcripts of *Camptotheca acuminata*, *Catharanthus roseus* and *Rauvolfia serpentina* transcriptomes. A. Molecular function, B. Biological process, C. Cellular Component.
(PDF)

Table S1 **Tissue/treatment description for RNA samples used in this study.**
(XLSX)

Table S2 **Expression abundances reported as fragments per kb transcript per million mapped reads in** ***Camptotheca acuminata*** **.**
(XLSX)

Table S3 **Expression abundances reported as fragments per kb transcript per million mapped reads in** ***Catharanthus roseus*** **.**
(XLSX)

Table S4 **Expression abundances reported as fragments per kb transcript per million mapped reads in** ***Rauvolfia serpentina*** **.**
(XLSX)

Table S5 **List of transcripts with orthologs in all three species.**
(XLSX)

Table S6 **List of transcripts with orthologs only in** ***Catharanthus roseus*** **and** ***Rauvolfia serpentina*** **.**
(XLSX)

Table S7 **List of transcripts with orthologs only in** ***Catharanthus roseus*** **and** ***Camptotheca acuminata*** **.**
(XLSX)

Table S8 **List of transcripts with orthologs only in** ***Camptotheca acuminata*** **and** ***Rauvolfia serpentina*** **.**
(XLSX)

Table S9 **List of transcripts in paralogous groups restricted to** ***Camptotheca acuminata*** **.**
(XLSX)

Table S10 **List of transcripts in paralogous groups restricted to** ***Catharanthus roseus*** **.**
(XLSX)

Table S11 **List of transcripts in paralogous groups restricted to** ***Rauvolfia serpentina*** **.**
(XLSX)
