# Supplementary material for: Development of Transcriptomic Resources for Interrogating the Biosynthesis of Monoterpene Indole Alkaloids in Medicinal Plant Species
Source: PLoS One. 2012 Dec 26;7(12):e52506. doi: 10.1371/journal.pone.0052506 (PMC3530497; doi:10.1371/journal.pone.0052506)
Supplement: Figure S1 — Gene Ontology functional annotation. Assignment of Gene Ontology Slim (GOSlim) terms to the Arabidopsis thaliana proteome, and the representative transcripts of Camptotheca acuminata, Catharanthus roseus and Rauvolfia serpentina transcriptomes. A. Molecular function, B. Biological process, C. Cellular Component. (PDF) [file pone.0052506.s001.pdf]

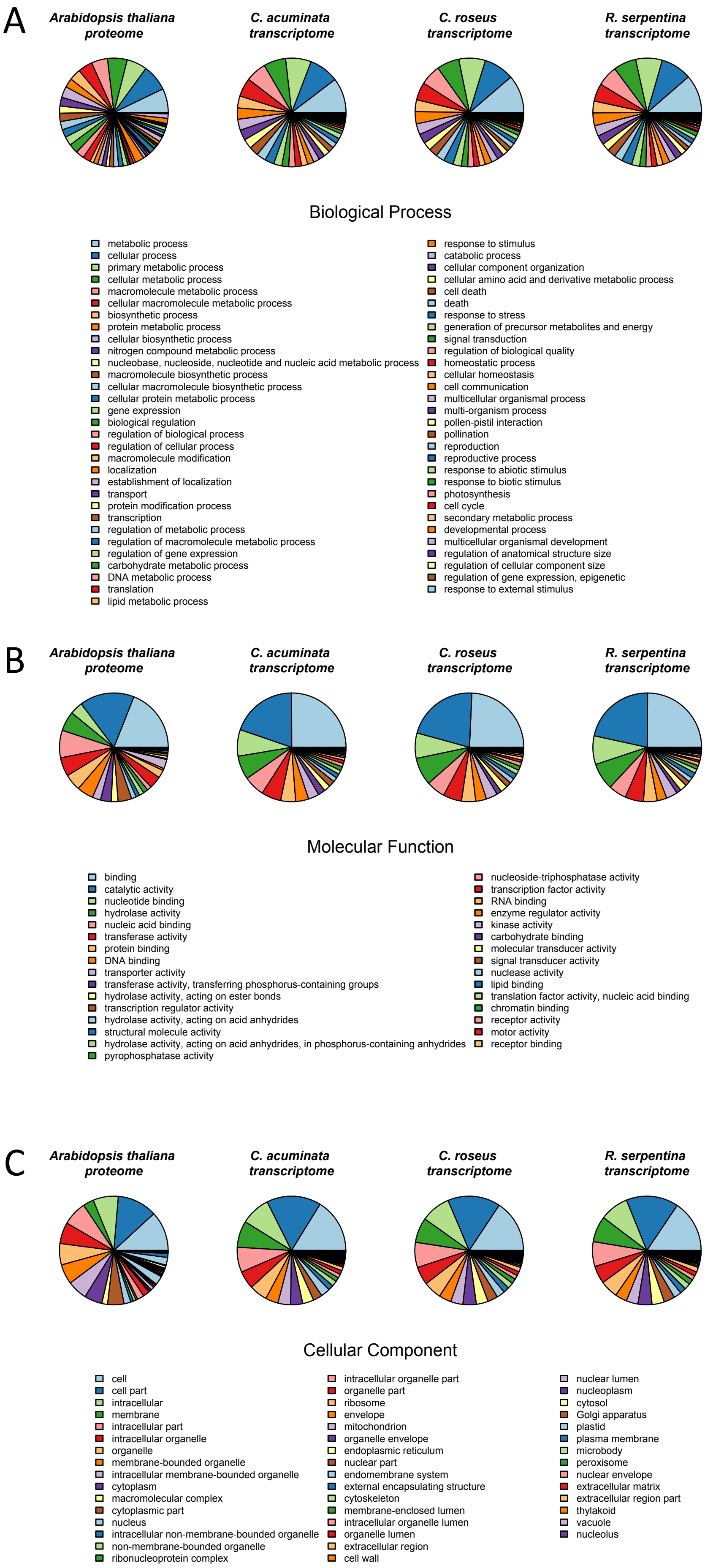
A
Arabidopsis thaliana proteome
C. acuminata transcriptome
C. roseus transcriptome
R. serpentina transcriptome
Biological Process
metabolic process
cellular process
primary metabolic process
cellular metabolic process
macromolecule metabolic process
cellular macromolecule metabolic process
biosynthetic process
protein metabolic process
cellular biosynthetic process
nitrogen compound metabolic process
nucleobase, nucleoside, nucleotide and nucleic acid metabolic process
macromolecule biosynthetic process
cellular macromolecule biosynthetic process
cellular protein metabolic process
gene expression
biological regulation
regulation of biological process
regulation of cellular process
macromolecule modification
localization
establishment of localization
transport
protein modification process
transcription
regulation of metabolic process
regulation of macromolecule metabolic process
regulation of gene expression
carbohydrate metabolic process
DNA metabolic process
translation
lipid metabolic process
response to stimulus
catabolic process
cellular component organization
cellular amino acid and derivative metabolic process
cell death
death
response to stress
generation of precursor metabolites and energy
signal transduction
regulation of biological quality
homeostatic process
cellular homeostasis
cell communication
multicellular organismal process
multi-organism process
pollen-pistil interaction
pollination
reproduction
reproductive process
response to abiotic stimulus
response to biotic stimulus
photosynthesis
cell cycle
secondary metabolic process
developmental process
multicellular organismal development
regulation of anatomical structure size
regulation of cellular component size
regulation of gene expression, epigenetic
response to external stimulus
B
Arabidopsis thaliana proteome
C. acuminata transcriptome
C. roseus transcriptome
R. serpentina transcriptome
Molecular Function
binding
catalytic activity
nucleotide binding
hydrolase activity
nucleic acid binding
transferase activity
protein binding
DNA binding
transporter activity
transferase activity, transferring phosphorus-containing groups
hydrolase activity, acting on ester bonds
transcription regulator activity
hydrolase activity, acting on acid anhydrides
structural molecule activity
hydrolase activity, acting on acid anhydrides, in phosphorus-containing anhydrides
pyrophosphatase activity
nucleoside-triphosphatase activity
transcription factor activity
RNA binding
enzyme regulator activity
kinase activity
carbohydrate binding
molecular transducer activity
signal transducer activity
nuclease activity
lipid binding
translation factor activity, nucleic acid binding
chromatin binding
receptor activity
motor activity
receptor binding
C
Arabidopsis thaliana proteome
C. acuminata transcriptome
C. roseus transcriptome
R. serpentina transcriptome
Cellular Component
cell
cell part
intracellular
membrane
intracellular part
intracellular organelle
organelle
membrane-bounded organelle
intracellular membrane-bounded organelle
cytoplasm
macromolecular complex
cytoplasmic part
nucleus
intracellular non-membrane-bounded organelle
non-membrane-bounded organelle
ribonucleoprotein complex
intracellular organelle part
organelle part
ribosome
envelope
mitochondrion
organelle envelope
endoplasmic reticulum
nuclear part
endomembrane system
external encapsulating structure
cytoskeleton
membrane-enclosed lumen
intracellular organelle lumen
organelle lumen
extracellular region
cell wall
nuclear lumen
nucleoplasm
cytosol
Golgi apparatus
plastid
plasma membrane
microbody
peroxisome
nuclear envelope
extracellular matrix
extracellular region part
thylakoid
vacuole
nucleolus
